# Proteomic Profiles Associated With Postsurgical Progression in Nonfunctioning Pituitary Adenomas

**DOI:** 10.1210/clinem/dgad767

**Published:** 2023-12-29

**Authors:** Tobias Hallén, Gudmundur Johannsson, Annika Thorsell, Daniel S Olsson, Charlotte Örndal, Angelica Engvall, Frida Jacobson, Anna Widgren, Jonas Bergquist, Thomas Skoglund

**Affiliations:** Department of Neurosurgery, Sahlgrenska University Hospital, 412 65 Gothenburg, Sweden; Department of Clinical Neuroscience, Institute of Neuroscience and Physiology, Sahlgrenska Academy, University of Gothenburg, 405 30 Gothenburg, Sweden; Department of Endocrinology, Sahlgrenska University Hospital, 413 46 Gothenburg, Sweden; Department of Internal Medicine and Clinical Nutrition, Institute of Medicine, Sahlgrenska Academy, University of Gothenburg, 405 30 Gothenburg, Sweden; Proteomics Core Facility at Sahlgrenska Academy, Gothenburg University, 413 90 Gothenburg, Sweden; Department of Endocrinology, Sahlgrenska University Hospital, 413 46 Gothenburg, Sweden; Department of Internal Medicine and Clinical Nutrition, Institute of Medicine, Sahlgrenska Academy, University of Gothenburg, 405 30 Gothenburg, Sweden; Late-stage Clinical Development, Cardiovascular, Renal and Metabolism (CVRM), BioPharmaceuticals R&D, AstraZeneca, 431 83 Gothenburg, Sweden; Department of Pathology, Karolinska University Hospital, 171 76 Stockholm, Sweden; Department of Neuroradiology, Sahlgrenska University Hospital, 413 46 Gothenburg, Sweden; Proteomics Core Facility at Sahlgrenska Academy, Gothenburg University, 413 90 Gothenburg, Sweden; Department of Chemistry–BMC, Analytical Chemistry and Neurochemistry, Uppsala University, 75124 Uppsala, Sweden; Department of Chemistry–BMC, Analytical Chemistry and Neurochemistry, Uppsala University, 75124 Uppsala, Sweden; Department of Neurosurgery, Sahlgrenska University Hospital, 412 65 Gothenburg, Sweden; Department of Clinical Neuroscience, Institute of Neuroscience and Physiology, Sahlgrenska Academy, University of Gothenburg, 405 30 Gothenburg, Sweden

**Keywords:** quantitative proteomics, nonfunctioning pituitary adenoma, tumor progression, reintervention

## Abstract

**Context:**

There is a lack of reliable biomarkers capable of predicting postoperative tumor progression of nonfunctioning pituitary adenomas (NFPAs).

**Objective:**

To discover proteomic profiles associated with postoperative tumor progression in patients with NFPAs. This was a case-controlled exploratory study at a tertiary university hospital. Tissue samples were obtained from 46 patients with residual tumor following surgery for NFPAs of gonadotroph lineage. Two patient groups were compared: patients requiring reintervention due to residual tumor progression (cases; reintervention group, n = 29) and patients with a residual tumor showing no progression for a minimum of 5 years (controls; radiologically stable group, n = 17). Differentially expressed proteins (DEPs) between patient groups were measured.

**Results:**

Global quantitative proteomic analysis identified 4074 proteins, of which 550 were differentially expressed between the 2 groups (fold change >80%, false discovery rate–adjusted *P* ≤ .05). Principal component analysis showed good separation between the 2 groups. Functional enrichment analysis of the DEPs indicated processes involving translation, ROBO-receptor signaling, energy metabolism, mRNA metabolism, and RNA splicing. Several upregulated proteins in the reintervention group, including SNRPD1, SRSF10, SWAP-70, and PSMB1, are associated with tumor progression in other cancer types.

**Conclusion:**

This is the first exploratory study analyzing proteomic profiles as markers of postoperative tumor progression in NFPAs. The findings clearly showed different profiles between tumors with indolent postoperative behavior and those with postoperative tumor progression. Both enriched pathways involving DEPs and specific upregulated proteins have previously been associated with tumor aggressiveness. These results suggest the value of proteomic profiling for predicting tumor progression in patients with NFPAs.

Pituitary tumors are mainly benign adenomas, with nonfunctioning pituitary adenomas (NFPAs) being the most prevalent. Residual tumor after surgery of NFPAs is common and described in up to half of patients after primary pituitary adenoma surgery (1). Although the tumors are frequently histologically benign, the clinical course of residual tumors varies greatly. Progression of a residual tumor is reported in 30% to 50% of cases (2, 3), and postoperative tumor progression is associated with increased mortality (4). However, the molecular mechanisms underlying the different clinical courses remain unknown, and there are currently no reliable prognostic markers capable of predicting the clinical course of a residual tumor (5).

Proteomic techniques have been used effectively in cancer research to identify clinically applicable biomarkers and therapeutic targets (6). Additionally, these methods have allowed comparisons of pituitary adenoma tissue with normal pituitary tissue (7) in order to identify adenoma subtypes (8-10) and proteins associated with their invasiveness (11-14). However, to the best of our knowledge, comparative proteomics have not been employed to identify proteomic profiles capable of predicting the postoperative tumor progression of NFPAs.

In this study, we evaluated the differences in NFPA-specific proteomic profiles between patients with a residual tumor requiring reintervention due to postoperative progression and patients with a residual tumor without progression following at least 5 years of postsurgery follow-up. We employed a case–control study design, including patient groups with distinctly different clinical courses and homogenous tumor material comprising only NFPAs of gonadotroph lineage, to increase the likelihood of identifying differences in proteomic profiles attributed to tumor progression.

## Material and Methods

### Patients

The patient cohort used in this study was the same as that used in a previous study investigating DNA methylation patterns associated with postoperative tumor progression in patients with NFPAs (15). The Swedish National Patient Registry and a database of surgeries performed at the Department of Neurosurgery, Sahlgrenska University Hospital, were used to identify patients surgically treated for NFPAs within the western region of Sweden (Västra Götalandsregionen) between 1987 and 2014. Sahlgrenska University Hospital is the only provider of pituitary surgery in the western region of Sweden (population: ∼1.9 million people).

We identified a total of 340 patients who underwent primary pituitary surgery between 1987 and 2014 for NFPAs. Medical records were systematically reviewed for information, including clinical symptoms, tumor progression, hormone replacement therapy, and clinical course. Patients were followed until 2019, confirming a follow-up period of at least 5 years.

To investigate proteomic profiles associated with clinically significant postoperative tumor progression, we compared 2 different patient groups. The reintervention group (cases) comprised patients with postoperative tumor progression requiring reintervention, with reintervention defined as surgery or radiation therapy due to progression of a residual tumor. Exclusion criteria for this group were no residual tumor after primary surgery, reintervention of a stable tumor remnant, postoperative radiotherapy as part of the primary treatment, and postoperative tumor progression not requiring reintervention. The radiologically stable group (controls) comprised patients with a radiologically stable residual tumor during the course of at least a 5-year postsurgery follow-up period. Exclusion criteria for this group were no residual tumor after primary surgery and postoperative radiotherapy as part of the primary treatment.

**Figure 1.**
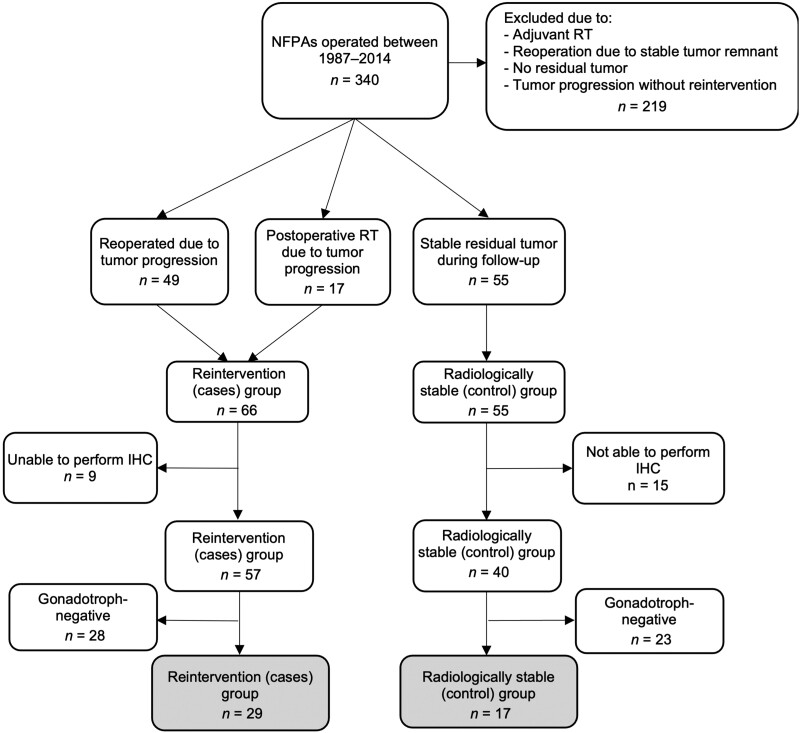
Flowchart describing the study inclusion and exclusion criteria for patients. IHC, immunohistochemistry; NFPA, nonfunctioning pituitary adenoma; RT, radiotherapy.

Of the 340 patients identified with an NFPA, 121 fulfilled the clinical inclusion criteria (66 required reintervention due to growth of a postoperative residual tumor [cases] and 55 had a stable tumor remnant for at least 5 years [controls]). To increase the homogeneity of the tumor material, only patients with NFPAs of gonadotroph lineage were included in the analysis. After excluding patients with nonsufficient tumor material, proteomic analysis was performed on 29 tumors from the cases group and 17 from the control group (Fig. 1).

### Ethics

The study was approved by the Regional Ethical Review Board in Gothenburg (Dnr: 100-15), Sweden, and by the National Board of Health and Welfare, Sweden.

### Immunohistochemistry Analysis

Immunohistochemistry (IHC) analysis was performed as previously described for adenoma tissue verification and tumor lineage classification (15). Immediately after surgery, tumor samples were fixed in 4% buffered formalin, and formalin-fixed paraffin-embedded (FFPE) tissue blocks were prepared and stored at the Department of Pathology, Sahlgrenska University Hospital. Sections (4 µm thick) were obtained from the FFPE blocks, with 1 slide stained with hematoxylin and eosin for adenoma tissue verification, and additional slides subjected to IHC staining using Agilent EnVision (Agilent Technologies, Santa Clara, CA, USA) or the Biocare detection system (Biocare Medical, Pacheco, CA, USA) according to the manufacturer’s instructions. The following primary antibodies were used at the dilutions indicated: adrenocorticotropin (1:100; M3501/A2A3; Dako/Agilent RRID:AB_2166039; Agilent Technologies), growth hormone (1:200; A0570, Dako/Agilent RRID:AB_2617170; Agilent Technologies), prolactin (1:300; ab64377; RRID:AB_1142327; Abcam, Cambridge, UK), follicle-stimulating hormone (1:500; M3504/C10; Dako/Agilent RRID:AB_2079146; Agilent Technologies), luteinizing hormone; 1:50; M3502/C93; Dako/Agilent RRID:AB_2135325; Agilent Technologies), thyrotropin (1:50; M3503/0042; Dako/Agilent RRID:AB_2287785; Agilent Technologies), pituitary-specific positive transcription factor-1 (Pit-1; 1:100; anti-SLC20A1/HPA035834; RRID:AB_2674809; Atlas Antibodies, Bromma, Sweden, and T-box family member (TBX19/T-Pit; 1:200; RRID:AB_2732209; Atlas Antibodies).

Tumor subclassification was determined according to positivity for hormone markers and/or transcription factors: thyrotroph (thyrotropin, Pit-1), somatotroph (growth hormone, Pit-1), corticotroph (adrenocorticotropin, T-Pit), gonadotroph (luteinizing hormone/follicle-stimulating hormone), lactotroph (prolactin and Pit-1), plurihormonal (multiple combinations), and null cell (none). IHC for steroidogenic factor 1 was not possible due to insufficient archival tumor material. Adenomas with an inconclusive staining pattern that prevented adequate classification were categorized as “adenoma not otherwise specified.” Therefore, tumors that could be classified as either null cell or gonadotroph were placed into this category and excluded from the study.

### Radiology

We retrieved 3 imaging scans (magnetic resonance or computed tomography) for each patient: the preoperative scan, the first postoperative scan, and the scan before reoperation or radiation therapy for patients in the cases group or the latest scan available for those in the control group. Tumor size, invasiveness (Knosp grade ≥3), and compression of the optic chiasm and hypothalamus were assessed by a neuroradiologist. Tumor volume was calculated by the formula (length × width × height)/2. All patients in the control group underwent radiologic follow-up for at least 5 years, and those in the cases group until reintervention. For 93% of patients between groups, imaging scans were performed by magnetic resonance.

### Proteomic Analysis and Protein Quantification

#### Deparaffinization of tissue samples

For each sample, new 20-μm-thick slices (n = 3) from FFPE tissue blocks were placed in Eppendorf tubes and deparaffinized according to previously described methods (16). Briefly, excess paraffin was carefully removed from tissue sections using a razor blade on a glass slide, followed by transfer to Eppendorf tubes for 4 rounds of incubation with 700 μL of xylene for 10 minutes. After incubation, the samples were centrifuged at 10 000*g* for 2 minutes at room temperature, and the resultant supernatant was discarded. Heptane (1 mL) was then added to the tubes prior to incubation and agitation at room temperature for 30 minutes. Methanol (80 μL) was then added to create an organic–aqueous 2-liquid phase system, where the upper heptane layer was removed, and the remaining methanol was allowed to evaporate. Tissue sections were sequentially rehydrated using an ethanol series (v/v: 2 × 100%, 90%, 80%, and 70%), followed by a final wash with Milli-Q water for 2 minutes.

#### Protein extraction

Deparaffinized samples were incubated in 200 μL of lysis buffer (7 M urea, 2 M thiourea, 1 M ammonium bicarbonate, and protease and phosphatase inhibitor cocktails) at 95 °C for 30 minutes (with shaking at 450 rpm), followed by incubation at 60 °C for 2 hours. The samples were then sonicated with a 3-mm sonication probe (30 1-second pulses at 30% amplitude), and tissue lysates were clarified by centrifugation for 10 minutes at 16 000*g* at 4 °C.

Total protein concentration in the samples was measured using the Bradford assay with bovine serum albumin as the standard (BioRad). Aliquots (30 μg) were obtained for digestion experiments.

#### Digestion

Proteins were reduced, alkylated, and on-filter digested using a multienzyme digestion with LysC and trypsin on Microcon-10 kDa centrifugal filters (Millipore, Dublin, Ireland) according to manufacturer instructions. The collected peptide filtrate was vacuum centrifuged to dryness using a speedvac system. The samples were then purified using Pierce C18 spin columns (Thermo Fisher Scientific, Waltham, MA, USA) and dried. Peptides were resolved in 90 μL of 0.1% formic acid and further diluted (4×) prior to nano liquid chromatography tandem mass spectrometry.

#### Liquid chromatography tandem mass spectrometry analysis

Samples were analyzed using a Q Exactive Plus Orbitrap mass spectrometer (Thermo Fisher Scientific, Bremen, Germany) equipped with a nano-electrospray ion source. Peptides were separated by reversed-phase liquid chromatography using an EASY-nLC 1000 system (Thermo Fisher Scientific) using a precolumn (EASY-column 100 μm × 2 cm, 5 μm C18 Thermo Fisher Scientific) and analytical column (EASY-column 75 μm × 15 cm, 3 μm, C18, Thermo Fisher Scientific) and eluted with a linear gradient from 4% to 100% acetonitrile at 250 nL/minute over the course of 150 minutes. The mass spectrometer was operated in positive ion mode and acquired a survey mass spectrum at a resolving power of 70 000 full width at half maximum (m/z = 400-1750 using an automatic gain control target of 3 × 10^6^). The 10 most intense ions were selected for higher-energy collisional dissociation fragmentation (25% normalized collision energy), with tandem mass spectrometry spectra generated with an automatic gain control target of 5 × 10^5^ at a resolution of 17 500. The mass spectrometer worked in data-dependent mode.

#### Database matching and label-free protein quantification

Identification and relative quantification were performed using Proteome Discoverer (v.2.4; Thermo Fisher Scientific) and the Mascot search engine (v.2.5.1; Matrix Science, London, UK), with matching performed using the SwissProt *Homo sapiens* database and tolerances of 5 ppm and 75 mmu for the precursor peptide and fragment, respectively. Tryptic peptides were accepted with 1 missed cleavage, variable methionine oxidation, and fixed cysteine alkylation. Only uniquely identified peptides were considered for relative quantification, and samples were normalized according to the total peptide count. Quantified proteins were filtered at a <5% false discovery rate (FDR) and grouped by sharing the same sequences to minimize redundancy. Sample ratios were calculated by dividing individual abundances by the group mean of the radiologically stable group. Fold change (FC) was determined by dividing the average for the reintervention group by that for the radiologically stable group.

### Statistical and Enrichment Analysis

Patient characteristics were compared between groups using the Mann–Whitney U test or Student's t test for continuous variables. Fisher's exact test was used to compare categorical variables. Data from proteomic analysis were further processed using Perseus (v.1.6.10.45; https://maxquant.net/perseus/) to determine significant differences in protein quantity according to Student's t test on log_2_ protein ratios, followed by Benjamini–Hochberg correction for multiple testing. Principal component analysis (PCA) was performed for the 2 patient groups in cases where proteins showed a maximum of 30% missing values. Proteins were considered differentially expressed when they were present in at least 70% of the patients and showed an FDR-adjusted *P* ≤ .05 and an average FC >80%. Heatmaps and volcano plots were generated in R (https://www.r-project.org/) and Rstudio (https://posit.co/download/rstudio-desktop/) using the packages pheatmap (https://cran.r-project.org/web/packages/pheatmap/index.html) and EnhancedVolcano (https://bioconductor.org/packages/release/bioc/html/EnhancedVolcano.html), respectively. For the heatmaps, missing values were imputed using the mean value of the existing values in the group.

### Pathway Enrichment Analysis

Pathway enrichment analysis was performed by mapping the 550 proteins showing significantly altered expression to biological pathways using the enrichPathway (https://www.rdocumentation.org/packages/ReactomePA/versions/1.16.2/topics/enrichPathway) package in R to generate a dot plot for the results (FDR-adjusted *P* ≤ .05). Gene Ontology (GO) annotations (November 2021) downloaded from the Uniprot database (https://www.uniprot.org/) were used to identify differentially regulated biological processes by using the enrichGO function in the clusterProfiler (https://bioconductor.org/packages/release/bioc/html/clusterProfiler.html) package of R to generate a dot plot for the results (FDR-adjusted *P* ≤ .05).

### Prediction of Protein–Protein Interactions

We employed STRING (v.11.5; http://string-db.org/cgi/input.pl) using the Markov clustering algorithm to construct protein–protein interaction (PPI) networks based on all differentially expressed proteins (DEPs) and for proteins showing upregulated expression in each respective patient group.

## Results

The case and control groups included 29 and 17 patients, respectively. Patients in the cases group were younger than those in the control group (55 vs 64 years; *P* = .011). There were no significant differences in preoperative tumor volume, Knosp grade, or preoperative hormone deficiencies between groups (Table 1).

**Table 1.** Baseline characteristics of the patients with nonfunctioning pituitary adenoma included in the study

	Reintervention group (cases)	Radiologically stable group (controls)	*P^a^*
No.	29	17	
Age, y	55 (12)	64 (9)	.011
Gender, n (%)			1.0
Men	22 (76)	13 (76)	
Women	7 (24)	4 (24)	
Follow-up duration, months	106 (82, 138)	135 (101, 169)	.09
Time to reintervention, months	58 (32, 74)		
Preoperative hormone deficiencies*^b^*, n (%)			
Thyrotropin	10 (34)	7 (41)	.76
Adrenocorticotropin	6 (21)	7 (41)	.18
Sex steroids	2 (6)	4 (24)	.17
Diabetes insipidus	—	—	—
Tumor volume, cm^3^*^c^*	11.6 (9.1, 15.1)	10.5 (8.8, 18.4)	.59
Reaches the chiasm, n (%)	27 (100)	12 (92)	.33
Invasive (Knosp grade ≥3), n (%)	11 (42)	5 (39)	1.0

Data are shown as the mean ± SD or median (25th percentile, 75th percentile).

^*a*^Between-group comparisons were performed using Fisher's exact test and Student's t test or the Mann–Whitney U test for categorical and continuous variables, respectively.

^*b*^None of the patients were tested preoperatively for growth hormone deficiency.

^*c*^Preoperative tumor volume.

### Identification of DEPs

**Figure 2.**
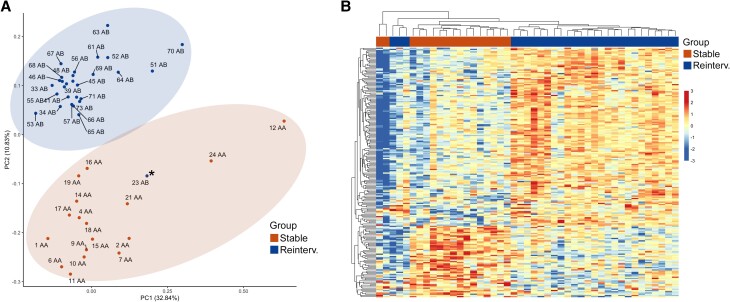
(A) PCA of radiologically stable tumors (AA) and tumors requiring reintervention (AB) based on their proteomic expression profiles showed a clear separation of the 2 patient groups, except for 1 patient (23AB; asterisk). The variation was explained by component 1 (32.8%) and component 2 (10.8%). (B) Unsupervised hierarchical clustering of significantly upregulated proteins (FC > 80%; FDR-adjusted *P* ≤ .05) showed 2 main clusters corresponding well to the control group (15 tumors, stable) and the cases group (25 tumors, reintervention), except for 5 patients comprising patients from both groups. FC, fold change; FDR, false discovery rate; PCA, principal component analysis.

Global quantitative proteomic analysis identified a total of 4075 proteins, with 3205 proteins considered for quantitative evaluation according to a low percentage (<30%) of missing values for each patient group. For an overall assessment of similarities and differences in proteomics data between groups, we performed PCA (Fig. 2A), which revealed clearly separate clusters between patient groups, except for 1 patient from the cases group who was included in the control cluster. Because we were unable to identify a reason for this exception according to clinical data, sample collection, or analytical method, we considered this patient to be an outlier and was excluded from further analyses.

Statistical analysis of the quantified proteins revealed 550 DEPs (FC > 80%, FDR adjusted *P* ≤ .05). Unsupervised hierarchical clustering of these proteins to gain a functional overview of differences revealed 2 main clusters corresponding well with the patient groups visualized in a heat map, except for 5 patients (Fig. 2B). These results agreed with the PCA plot, where the same 5 patients (3 from the cases group and 2 from the control group) did not group tightly with the other individuals in their respective groups.

**Figure 3.**
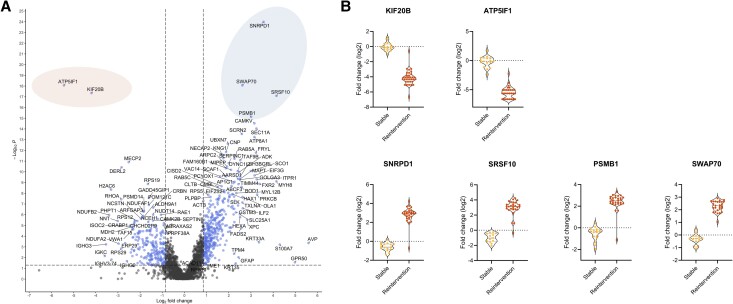
(A) Volcano plot of *P* vs log_2_ FC in radiologically stable tumors (left) compared with tumors requiring reintervention (right). Significantly upregulated and downregulated proteins are highlighted (in blue) (FC > 80%; *P* ≤ .05). Proteins within ellipses are described in the text. (B) Violin plot presenting proteins with the highest degree of differential expression between study groups. FC, fold change.

We then performed comparative analysis to identify and visualize DEPs between the 2 groups in a volcano plot (Fig. 3A). The results showed significant higher expression levels for 188 proteins in the control group and 362 in the cases group. Among the most upregulated proteins in the cases group were small nuclear ribonucleoprotein D1 (SNRPD1), serine/arginine splicing factor 10 (SRSF10), switch-associated protein 70 (SWAP70), and proteasome 20S subunit β1 (PSMB1), as well as kinesin family member 20B (KIF20B) and ATP synthase inhibitory factor subunit 1 (ATP5IF1) in the control group (Fig. 3B).

### Pathway Enrichment Analysis of DEPs

**Figure 4.**
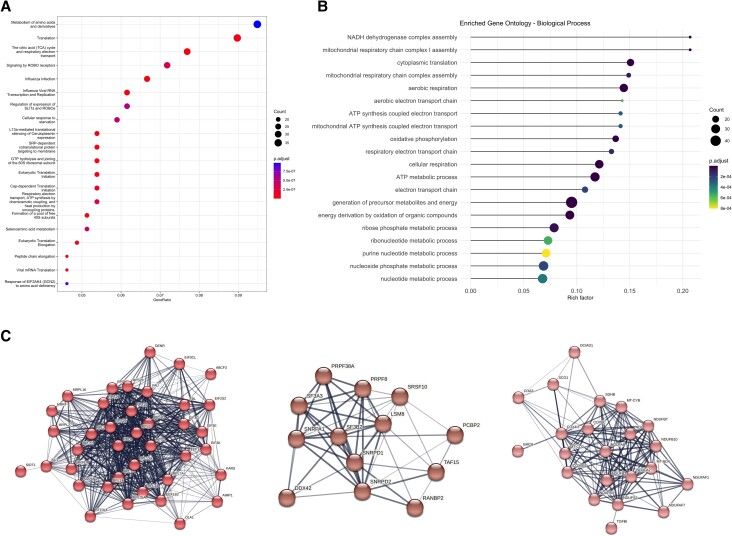
(A) Reactome pathway analysis displayed as a dot plot of the top 20 enriched pathways derived from the 550 proteins displaying significantly altered expression patterns (FC > 80%; FDR-adjusted *P* ≤ .05). The color of the dot designates the significance of the change in expression (adjusted *P*). The size of the dot signifies the number of enriched proteins. The X-axis represents the protein ratio (ie, the percentage of enriched target proteins among total proteins), and the Y-axis shows the name of the pathway. (B) Dot plot of the top 20 enriched Gene Ontology biological processes based on the 550 proteins showing significantly altered expression patterns (FC > 80%; FDR-adjusted *P* ≤ .05). The X-axis shows Rich factor and the color of the dot designating the significance of the change in expression (adjusted *P*). The size of the dot represents the number of proteins assigned to each biological process. (C) PPI and functional enrichment analysis for the 550 proteins showing significant differential expression revealed tight clusters of proteins associated with translation (left), RNA splicing (middle) and energy metabolism (right). FC, fold change; FDR, false discovery rate; PPI, protein–protein interaction.

Among the top biological pathways when analyzing all DEPs in Reactome were as follows: metabolism of amino acids and derivates, translation, citric acid cycle and respiratory electron transport, and signaling by ROBO receptors (Fig. 4A). The top biological processes according to GO terms from Uniprot included translation and energy metabolism such as mitochondrial respiration and ATP processing (Fig. 4B).

### Construction of a PPI Network

To reveal potentially biological relevant processes and pathways for the DEPs, we performed STRING analysis first using all 550 proteins and then separately for the upregulated proteins in the cases (n = 362) and control (n = 188) groups. Of the 550 proteins, 542 were available for STRING analysis, which revealed clusters corresponding to translation, RNA splicing, and energy metabolism (including oxidative phosphorylation and respiratory electron transport) (Fig. 4C). The same clusters were observed for 360 upregulated proteins available for STRING analysis in the cases group, and for the control group (n = 182 proteins available for analysis) also clusters corresponding to energy metabolism and translation were noted.

## Discussion

This is the first study to perform global proteomic analysis as a method to identify markers of postoperative tumor progression in patients with NFPAs. The analyses revealed profiles associated with clinically significant postoperative tumor growth. Interestingly, enriched biological pathways and specific upregulated proteins in the cases group have previously been identified as associated with tumor growth in various cancers.

Global proteomic analyses have been increasingly used during the previous decade to identify disease biomarkers associated with tumor growth (6, 17). To the best of our knowledge, this type of analysis has not been undertaken to identify and compare proteomic profiles associated with postoperative tumor progression of NFPAs.

In this study, PCA of all quantified proteins and hierarchical clustering analysis on DEPs visualized in a heat map revealed different profiles between tumors showing clinically significant postoperative progression and tumors that remained indolent after surgery (Fig. 2). This could indicate an important difference in the expression of specific proteins associated with increased risk of postoperative tumor progression.

To identify proteins associated with biological functions or pathways that are overrepresented among the DEPs, functional enrichment analysis was performed. In the reactome pathway analysis translation and signaling by ROBO receptors were among the top enriched pathways sharing several proteins (Fig. 4A). Protein–protein interaction and functional enrichment analysis using STRING also revealed tight clusters of proteins associated with translation, indicating a biological relevance of these proteins (Fig. 4C). ROBO transmembrane receptors interact with SLIT proteins and play important roles in axon guidance (18). However, previous studies also indicate that the ROBO/SLIT signaling pathway is involved with several cancer types, including glioblastoma (19), endometrial carcinoma (20), pancreatic cancer (21), and acute myeloid leukemia (22). Another tight cluster in the PPI analysis contained proteins involved with RNA splicing. Interestingly, proteins involved in mRNA splicing are among the most upregulated proteins in the reintervention group as indicated in the volcano and violin plots (Fig. 3). Splicing contributes to transcriptional diversification (23), and aberrant splicing has been implicated in various human cancers (23-25). Specifically, the spliceosome has been identified as a significant mechanistic driver of pituitary tumors (26), in which dysregulation of the splicing machinery has been demonstrated (27). Furthermore, signaling pathways related to the spliceosome are reportedly enriched in NFPAs (7). A common feature of several DEPs was their involvement as RNA binding proteins, with aberrant RNA binding protein expression previously being linked with different cancers (28, 29).

Other enriched biological processes included energy metabolism (Fig. 4B), which is a central feature of tumorigenesis (30, 31). The most enriched biological processes are involved in mitochondrial respiration, oxidative phosphorylation and ATP processes. This finding is in correlation with previous studies that identified mitochondrial dysfunction as a key factor in aberrant energy metabolism associated with pituitary tumor development (30, 32), as well as critical for the development of pituitary oncocytomas (33).

Upregulated proteins in the cases group may be potential biomarkers of a more aggressive phenotype in pituitary adenomas. Two of the most highly upregulated proteins in the cases group were RNA binding proteins involved in RNA splicing (Fig. 3). SNRPD1 is involved in pre-mRNA splicing and reportedly upregulated in several cancers, including lung adenocarcinoma and breast cancer (34, 35). Similarly, SRSF10 is involved in RNA splicing and as a regulator of oncogenesis in hepatocellular carcinoma, as well as cervical and colon cancer (36-38). Another upregulated protein was PSMB1, a regulator of mRNA stability, as well as cell growth, colony formation, and migration, and its upregulation was reported in esophageal cancer and ovarian cancer cell lines (39). Additionally, SWAP70, which is involved in cytoskeletal rearrangement, plays an important role in the oncogenesis of glioma and prostate cancer (40-42) (Fig. 3).

In the control group, the 2 most highly expressed proteins were ATP5IF1 and KIF20B (Fig. 3). ATP5IF1 regulates mitochondrial homeostasis and has in endometrial cancer been shown to have increased expression in tumor tissue versus normal tissue, although in tumor patients, higher levels of ATP5IF1 were associated with better overall survival (43). This is in concordance with our findings showing higher expression in the control group with indolent adenomas. Elevated levels of KIF20B has been associated with tumorigenesis in different cancer types; however, higher levels of KIF20B were found in less aggressive subtypes of ovarian cancer relative to levels identified in the most aggressive form (44), which is similar to our findings with higher levels in the control group.

This study has several limitations. These include the relatively small size of the study groups and the limited population from which the patients originated, both of which might introduce bias. Additionally, some adenomas of the gonadotroph subtype could have been excluded based on the unavailability of IHC for steroidogenic factor 1. Moreover, recent studies suggest that the current definition of the gonadotroph lineage might be insufficient (10, 45), suggesting that some heterogeneity within our tumor samples cannot be ruled out. Importantly, this study is exploratory, and additional investigations to confirm these findings are required. In future validation studies certain methodology features would be desirable: including a larger unselected cohort; tumor progression measurement by 3-dimensional volumetric segmentation to analyze the correlation between growth rate and specific proteomic markers; validation of markers with IHC and/or Western blot. However, the strengths of the study include the design enabling comparison of 2 distinct and clinically disparate patient groups and the inclusion of only 1 adenoma subtype.

In conclusion, this study demonstrates proteomic profiling of pituitary adenomas as a means of identifying biomarkers associated with postoperative tumor progression of NFPAs. These findings also provide a step forward in broadening our understanding of the mechanisms involved in tumor progression of NFPAs. Our future work will focus on further identification and validation of proteomic signatures and biomarkers predictive of NFPA-specific postsurgery tumor progression.

## Data Availability

Some or all datasets generated during and/or analyzed during the current study are not publicly available but are available from the corresponding author on reasonable request.

## Abbreviations

DEP: differentially expressed protein
FC: fold change
FDR: false discovery rate
FFPE: formalin-fixed paraffin-embedded
GO: Gene Ontology
IHC: immunohistochemistry
NFPA: nonfunctioning pituitary adenoma
PCA: principal component analysis
PPI: protein–protein interaction
